# Physicochemical and nanomedicine applications of phyto-reduced erbium oxide (Er_2_O_3_) nanoparticles

**DOI:** 10.1186/s13568-023-01527-w

**Published:** 2023-02-25

**Authors:** Hamza Elsayed Ahmad Mohamed, Ali Talha Khalil, Khaoula Hkiri, Muhammad Ayaz, Jamil Anwar Abbasi, Abdul Sadiq, Farhat Ullah, Asif Nawaz, Ikram Ullah, Malik Maaza

**Affiliations:** 1 UNESCO UNISA Africa Chair in Nanoscience and Nanotechnology, College of Graduate Studies, University of South Africa, Pretoria, South Africa; 2 Nanoscience African Network (NANOAFNET), Materials Research Department, iThemba LABS, Cape Town, South Africa; 3 Department of Pathology, Lady Reading Hospital Medical Teaching Institution, Peshawar, 25000 KP Pakistan; 4 Department of Pharmacy, Faculty of Biological Sciences, University of Malakand, Dir (L), KPK, Chakdara, 18000 Pakistan; 5 Department of Biotechnology & Genetic Engineering, Hazara University Mansehra, Mansehra, KP Pakistan

**Keywords:** Er_2_O_3_, Green nanotechnology, Diabetes, α-glucosidase and α-amylase

## Abstract

*Hyphaene thebaica* fruits were used for the fabrication of spherical erbium oxide nanoparticles (HT-Er_2_O_3_ NP_S_) using a one-step simple bioreduction process. XRD pattern revealed a highly crystalline and pure phase with crystallite size of ~ 7.5 nm, whereas, the W–H plot revealed crystallite size of 11 nm. FTIR spectra revealed characteristic Er-O atomic vibrations in the fingerprint region. Bandgap was obtained as 5.25 eV using K-M function. The physicochemical and morphological nature was established using Raman spectroscopy, reflectance spectroscopy, SAED and HR-TEM. HT-Er_2_O_3_ NP_S_ were further evaluated for antidiabetic potential in mice using in-vivo and in-vitro bioassays. The synthesized HT-Er_2_O_3_ NP_S_ were screened for in vitro anti-diabetic potentials against *α*-glucosidase enzyme and *α*-amylase enzyme and their antioxidant potential was evaluated using DPPH free radical assay. A dose dependent inhibition was obtained against *α*-glucosidase (IC_50_ 12 μg/mL) and *α*-amylase (IC_50_ 78 μg/mL) while good DPPH free radical scavenging potential (IC_50_ 78 μg mL^−1^) is reported. At 1000 μg/mL, the HT-Er_2_O_3_ NP_S_ revealed 90.30% and 92.30% inhibition of *α*-amylase and *α*-glucosidase enzymes. HT-Er_2_O_3_ NPs treated groups were observed to have better glycemic control in diabetic animals (503.66 ± 5.92*** on day 0 and 185.66 ± 2.60*** on day 21) when compared with positive control glibenclamide treated group. Further, HT-Er_2_O_3_ NP_S_ therapy for 21 days caused a considerable effect on serum total lipids, cholesterol, triglycerides, HDL and LDL as compared to untreated diabetic group. In conclusion, our preliminary findings on HT-Er_2_O_3_ NP_S_ revealed considerable antidiabetic potential and thus can be an effective candidate for controlling the post-prandial hyperglycemia. However, further studies are encouraged especially taking into consideration the toxicity aspects of the nanomaterial.

## Introduction

Advances in nanobiotechnology over the past few years have yielded exciting applications across different fields, especially in medicine and drug delivery (Chittaranjan Patra 2021; Khalil et al. 2021a). It has been well established that materials on the nanometer scale possess a unique surface area to volume ratio that provide peculiar characteristics like enhanced reactivity and higher efficacy (Mohamed et al. 2020a). Among the metallic nanoparticles; silver, gold, zinc, iron etc. are well studied for their biomedical and nanomedicine applications, however, the lanthanides rare earth oxides are not well-explored (Ovais et al. 2019, 2020). The lanthanides rare earth oxides are known for their distinctive features and therefore used in number of applications such as energy, catalysis, photonics, environment, solid state optoelectronics, telecommunications, solar cells etc. Nano rare earth oxides are anticipated as exciting materials attributed to the quantum confinement and shape specific attributes (Rahimi-Nasrabadi et al. 2017). Mostly research on rare earth oxides is usually centered on the evaluation of physical, electrical, optical properties (Diallo et al. 2016; Rajaji et al. 2019). Among them, trivalent erbium oxide nanoparticles (Er_2_O_3_ NPs) are considered as one of the fascinating materials for applications in physical and chemical sciences. It usually has a pinkish color and cubic structure. Er_2_O_3_ is known for its mechanical properties, stiffness, thermal and chemical durability, inertness and corrosion resistance. Mechanical strength of the Er_2_O_3_ is comparable to alumina and magnesia. Er_2_O_3_ is a wide bandgap material i.e., ~ 5.4 eV (Acikgoz et al. 2021; Azad and Maqsood 2014; Rajaji et al. 2019). Er_2_O_3_ nanomaterials have revealed excellent potential in applications related to the gas sensors, optical communication, phosphorus display monitors, imaging and photoelectrochemical water splitting materials (Mohammadi and Fray 2009; Radziuk et al. 2011). Previously, antibacterial activity of Er_2_O_3_ is also reported against different pathogens like *P. aeruginosa, E. faecalis, S. aureus* and *E. coli* (Dědková et al. 2017).

Various chemical and physical processes can be utilized for preparation of the metal oxide nanoparticles such as hydrolysis, hydrothermal, sol–gel, precipitation, pyrolysis, thermal decomposition, ball milling etc. Previously, Er_2_O_3_ NPs were synthesized using chemical precursor like erbium nitrate (Azad and Maqsood 2014). Other methods like chemical bath deposition, microemulsion, sonochemical synthesis, low-pressure metallorganic chemical vapor deposition (MOCVD), thermal decomposition, solvo-hydrothermal, sol–gel, radio frequency (RF) sputtering etc. have been published for the preparation of Er_2_O_3_ NPs (Losurdo et al. 2007; Nguyen et al. 2010; Pacio et al. 2021; Que et al. 2001; Rajaji et al. 2019; Tabanli et al. 2017). Hitherto, being effective, these processes have disadvantages such as the wet chemistry-based approaches can generate toxic waste while, the physical methods are energy intensive. Furthermore, nanoparticles synthesized using chemical approaches can have low biocompatibility that limits their applications in medicine (Ovais et al. 2021; Sani et al. 2021). Contrary to physico-chemical methods, biological synthesis provides a suitable, one-step and economical alternative to other conventional means. Biological materials like the extracts of medicinal plants can be used as reducing, stabilizing and capping agents for the preparation of metal-based NPs (Khalil et al. 2021b; Nasar et al. 2022).

Diabetes mellitus (DM), disorder of the glucose metabolism and associated with hyperglycemia as well as other complications like neuropathy, retinopathy, nephropathy, and micro and macrovascular complications (Arky 1982). Defects in the production, secretion and action of insulin are responsible for hyperglycemia (Booth et al. 2016). DM affects about five percent population worldwide and its occurrence is increasing at an alarming rate, and is also associated with number of other diseases (Rahim et al. 2019). There are about 450 million peoples around the world which are effected by DM which is estimated to effect 690 million people till 2044 (Cho et al. 2018). Broadly the disease has two types including Type-1 diabetes (T1DM) and Type-2 diabetes (T2DM) (Del Prete et al. 1977; Himsworth and Kerr 1939). Inhibitors of two vital enzymes including alpha-glucosidase and alpha-amylase implicated in gastrointestinal glucose absorption are considered as vital drug targets. These enzymes breakdown polysaccharides (starch) to glucose and the inhibition of these enzymes play an important role to reduce glucose absorption in the intestine (Gin and Rigalleau 2000). Beside these targets, scavenging excessive number of free radicals is important. These free radicals generated during metabolic processes are responsible for a series of human illnesses including atherosclerosis, immune system destruction, cancer, neurological disorders, heart disorders, cerebro-vascular diseases and metabolic disorders (Saeed et al. 2021). In DM, free radicals cause lipids peroxidation, glucose oxidation and glycation of proteins (non-enzymatic) leading to DM and its complications (Mir et al. 2019; Sadiq et al. 2020). Number of antioxidants which may be natural or synthetic are used and can be helpful in metabolic disorders management (Mahnashi et al. 2022b).

**Fig. 1:**
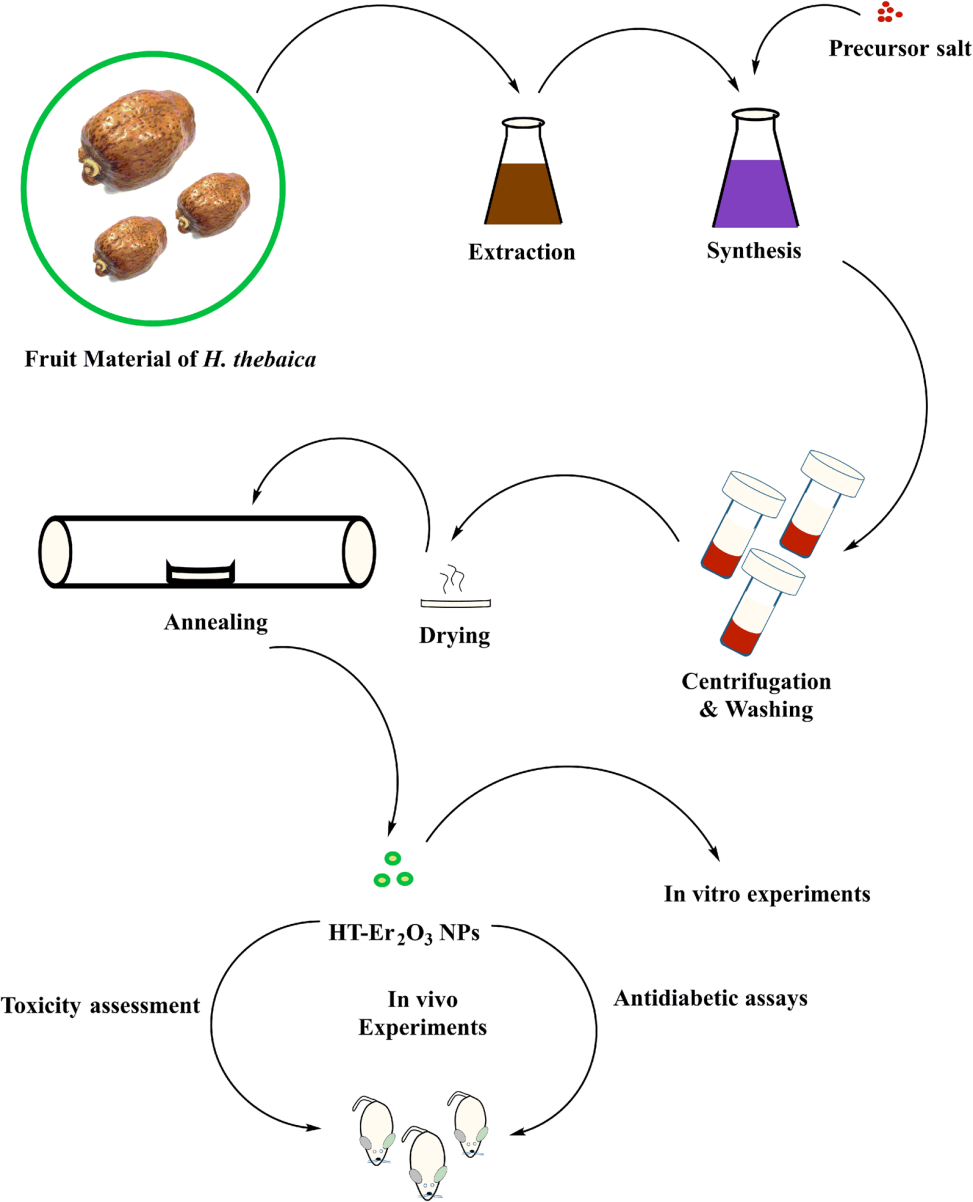
Graphical scheme of study

Herein, we have reported extracts modulated biosynthesis of HT-Er_2_O_3_ NPs for the 1st time by using plant extracts as bioreducing agents. Aqueous extracts of the edible fruit of *Hyphaene thebaica* (Egyptian Doum; gingerbread in English) was used as bioreducing agents. *H. thebaica* has well-established uses in the folkloric medicines and have been reported for dyslipidemia, hypertension, haematuria, bleeding, diuretic, diaphoretic, lowering blood pressure etc. (Abdulazeez et al. 2019; Khalil et al. 2018). After characterization different bioassays were performed (in-vitro & in-vivo) for determination of their antioxidant and antidiabetic potential & their safety was evaluated. Fig. 1 indicate the general study design for the present research.

## Materials and methods

### *H. thebaica* processing

The fruit material of *H. thebaica* was purchased in Aswan (Egypt), and was verified from a plant taxonomist. Fruit material was rinsed with double distilled water to clean any potential particulate material and kept for shade drying. Later, the fruit material grounded to fine powder and stored in zipper bags. Aqueous extraction was performed by heating 10 g powdered material in double distilled water (100 mL) for 1 h at 80 °C. Obtained mixture was cooled to room temperature and filtered three times for removing residual waste. The visually clear and transparent solution was used for synthesis.

### Biosynthesis of HT-Er_2_O_3_ NPs

For biosynthesis, the precursor salt i.e. erbium nitrate (Er(NO_3_)_3_) (4 g) was introduced to 100 mL extract solution and allowed to stand room temperature for few minutes. Later, the solution was centrifuged at 8000 RPM for 20 min, and the pelleted precipitates were washed thrice in distil water. The washed precipitates were dried for 2 h (at 100 °C). Resultant powdered material (assumed as HT-Er_2_O_3_ NPs) was placed in the ceramic boat and calcinated for 2 h (500 °C) in a tube furnace.

### Characterization

The physicochemical characteristics of the as synthesized NPs were analyzed via various techniques. XRD pattern was obtained to study the crystalline properties of HT-Er_2_O_3_ NPs. The x-ray diffractometer is equipped with Cu Kα (1.54 Å) radiation and the system is operating in Bragg–Brentano geometry The diffraction pattern was compared with the standard crystallography database and the crystallographic reflections were utilized to measure the average grain or crystallite size using Debye Scherrer approximation (Eq. 1) and Williamson-Hall equation (Eq. 2), as follows;

1 $$\langle D\rangle_{{{\text{SIZE}}}} = {\raise0.7ex\hbox{${K\lambda }$} \!\mathord{\left/ {\vphantom {{K\lambda } {\Delta \theta_{{{1 \mathord{\left/ {\vphantom {1 2}} \right. \kern-0pt} 2}}} }}}\right.\kern-0pt} \!\lower0.7ex\hbox{${\Delta \theta_{{{1 \mathord{\left/ {\vphantom {1 2}} \right. \kern-0pt} 2}}} }$}}$$

2 $$\beta \cos \theta = \left( {{\raise0.7ex\hbox{${K\lambda }$} \!\mathord{\left/ {\vphantom {{K\lambda } D}}\right.\kern-0pt} \!\lower0.7ex\hbox{$D$}}} \right) + 4\varepsilon \sin \theta .$$

here “λ” = 1.54 Å, and “K” = 0.9

The values of β cosθ were plotted as a function of 4 sinθ on the x-axis, followed by the linear fit for calculating the y-intercept and strain (ε) for obtaining the grain or crystallite size (D) using Williamson-Hall plot method (Zak et al. 2011). Furthermore, the dislocation density was determined using the formula (Bindu and Thomas 2014);

3 $$\delta = {\raise0.7ex\hbox{$1$} \!\mathord{\left/ {\vphantom {1 {D^{2} }}}\right.\kern-0pt} \!\lower0.7ex\hbox{${D^{2} }$}}$$

Where as; “δ” indicate dislocation density and “D” indicate grain or crystallite size of the nanoparticles.

The vibrational properties of the nanoparticles were studied by FTIR in the spectral range (4000 cm^−1^ to 400 cm^−1^) and Raman spectra at room temperature with laser line 473 nm and average excitation power of 2.48 mW. Reflectance spectra was obtained in the range of 200 nm to 800 nm, and was used to obtain the optical bandgap using Kubelka–Munk function (Guler et al. 2015). High Resolution Transmission Electron Microscopy (HR-TEM) was performed to study the nanoparticles shape and morphology. Particle size distribution was obtained after processing the images using Image J. SAED pattern (Selected Area Electron Diffraction Pattern) was also obtained.

### In-vitro experiments

#### Inhibition of alpha-glucosidase enzyme

The inhibitory potentials of Er_2_O_3_ NPs against alpha-glucosidase enzyme was evaluated according to protocol described previously with minor modification (Hussain et al. 2019). Commercially available enzymes were used in the study. Alpha-glucosidase (CAS 9001-42-7) used in the study was purchased from (sigma- Aldrich, USA). Glucosidase solution was prepared by dissolving α-glucosidase enzyme (0.5 µ/ml) in 120 µL of phosphate buffer with pH adjusted at 6.9. Buffer solution (0.1 M having pH 6.9) was used to prepare solution the enzyme substrate. Various serial samples dilutions were prepared ranging from 62.5 to 1000 μg mL^−1^ concentration were prepared. Initially, solution of enzyme was added to sample solution with subsequent incubation at 37 °C for 20 min. To this mixture, 20 μL of substrate solution was added and incubated again at the same temperature for fifteen minutes. To abort the reaction, 80 μL of Na_2_O_3_ solution was added to the mixture and the absorbance at 405 nm was recorded via spectrophotometer. Positive control was acarbose whereas, the blank one contained the same mixture without inhibitor agents. Finally, % α-glucosidase inhibitory results were calculated using our previously described procedure (Mahnashi et al. 2022b).

#### α-amylase inhibition

Α-amylase inhibitory studies of the selected nanoparticles were performed as per the previously established procedure (Nair et al. 2013). Alpha-amylase from human saliva, Type XIII-A, lyophilized powder, 300–1500 units/mg protein (CAS 9000-90-2, EC 232-565-6) was obtained from Merck KGaA, Darmstadt, Germany. Briefly, α-amylase enzyme (20 μL) and 0.02 M sodium phosphate buffer (200 μL) was added to the test samples of different concentrations (31.25–1000 μg mL^−1^), followed by 10 min incubation at room temperature and subsequent addition of starch (200 μl). 400 μL DNS (dinitrosalicylic acid) was added to stop the reaction, followed by boiling in water bath for 5 min and then allowed to cool. Finally, 15 mL double distilled water was introduced to the reaction mix and readings were recorded at 540 nm. No test sample was added the controls. Acarbose was utilized as standard α-amylase inhibitor.

#### DPPH-radicals scavenging assay

Anti-oxidant potential of HT-Er_2_O_3_ NPs (test compound) was determined by using 1,1-Diphenyl, 2-Picrylhydrazal (DPPH) (Dhanasekaran et al. 2015; Kamal et al. 2015). For making DPPH solution, 100 mL methanol was taken, to which 24 mg of DPPH and dissolved. Different dilutions of the test compound, ranging from 62.50 to 1000 μg mL^−1^ were prepared. One mL of DPPH solution was added to 1 mL of test sample dilution and subsequently incubated in dark place for 30 min at 23 °C. Subsequent to incubation, absorbance values were recorded at 517 nm via UV–visible spectrophotometer. The positive control was ascorbic acid, whereas the negative control was DPPH solution without test sample. Percent scavenging of test compounds was determined via formula;

$$\user2{\% FRSA} = \left( {\frac{{{\varvec{Ab}}_{{\varvec{C}}} - \user2{ Ab}_{{\varvec{S}}} }}{{{\varvec{Ab}}_{{\varvec{C}}} }}} \right) \times 100$$

### In vivo diabetic studies

#### Animals and approval of ethical committee

BALB/c albino mice were utilized for the anti-diabetic study. Animals were acquired from NIH Pakistan and looked after in the animal house of the University of Malakand. Animals were maintained under normal day/night cycle (12 h light and 12 h dark) and provided enough food and ad libitum. The procedure and project were approved by research ethics committee at the Department of Pharmacy, University of Malakand. All animals procedure and handling were performed following the Commission on life sciences, National research council 1996 (Council) (NRC 1996).

#### Acute toxicity test

To analyze the toxicological effects of our test sample, albino mice were classified into different groups (n = 5) and were administered i/p dose of 200–2000 mg kg^−1^ of our test compound. Animals were closed observed for one week for the appearance of any symptoms of toxicity including lethality or any aberrant behavior (Ayaz et al. 2017; Mir et al. 2019).

#### Induction of diabetes

For diabetes induction, mice were kept in fasting for 8–12 h and ten percent alloxan-monohydrate (150 mg kg^−1^) was administered intraperitoneally (I/P). Normal saline (NS) was given to control group. Following inducing of DM, blood glucose level was calibrated with glucose meter and only diabetic animals for further proceedings were proceeded (Hussain et al. 2019).

#### Experiment design

Mice were divided randomly into 4 groups and each group constituted of 5 mice. The concentration of glucose in blood was observed at 0th, 1st, 4th, 7th, 11th, 14th, and 21st days of treatment.

- Group I: Diabetic group was given alloxan and Tween 80 (I/P).
- Group II: Control nondiabetic group was given normal saline only (I/P).
- Group III: Treatment group was given glibenclamide (dose μg kg^−1^) PO.
- Group IV: Treatment group received test compound (dose μg kg^−1^) (I/P).

#### Oral glucose tolerance test (OGTT)

To evaluate the animal’s glucose tolerance capacity after oral glucose administration, animals of both groups including normal control and disease control were kept on fasting overnight. Subsequently, 2 mg/kg oral glucose was administered to each animal. Blood glucose was monitored at different times from (0 to120 min) after administration of glucose (Mahnashi et al. 2022a).

#### Drugs administration and assessment of blood glucose level

Animals were distributed in four groups. Group 1 was administered Alloxan at 150 mg kg^−1^ dose and tween 80 and acted as disease control group. This group was untreated and was kept for comparison with the treated groups. Group 2 was placebo group which was maintained on normal diet for comparison, Group 3 was disease group administered with standard drug glibenclamide at 5 mg/kg dose daily for 21 days. Further, group 4 was disease group which was maintained on 30 mg/kg dose of our test compound. Blood glucose levels were monitored three times a day using glucometer (Viva check Inox Laboratories Wilmington USA) and were observed for 21 days (Shaheen et al. 2016).

#### Blood biochemistry analysis

Animals were euthanized at the end of experiments, via halothane anesthesia following standard procedure and samples of blood from lateral tail were collected for further analysis. Blood tests including cholesterol level, triglycerides, LDL, HDL, and total lipids were analyzed via techno 786 semi-automated bio-chemistry analyzer. Colorimetric enzymatic test using glycerol-3-phosphate-oxidase (gpo) and Chod-Pap enzymatic photometric tests were employed for the analysis (Pundir and Aggarwal 2017).

### Statistical analysis

Experimental procedures were performed three times and the results are reported as Mean ± SEM. One way ANOVA and multiple comparison DUNNETT’s test was used for the statistical comparison of the treatment and control groups using Graph Pad Prism version 5.

### Estimation of median inhibitory concentrations

The dose–response curve was used to calculate the Median inhibitory concentrations (IC_50_) using using Graph Pad Prism version 5.

## Results

### Characterization

**Fig. 2:**
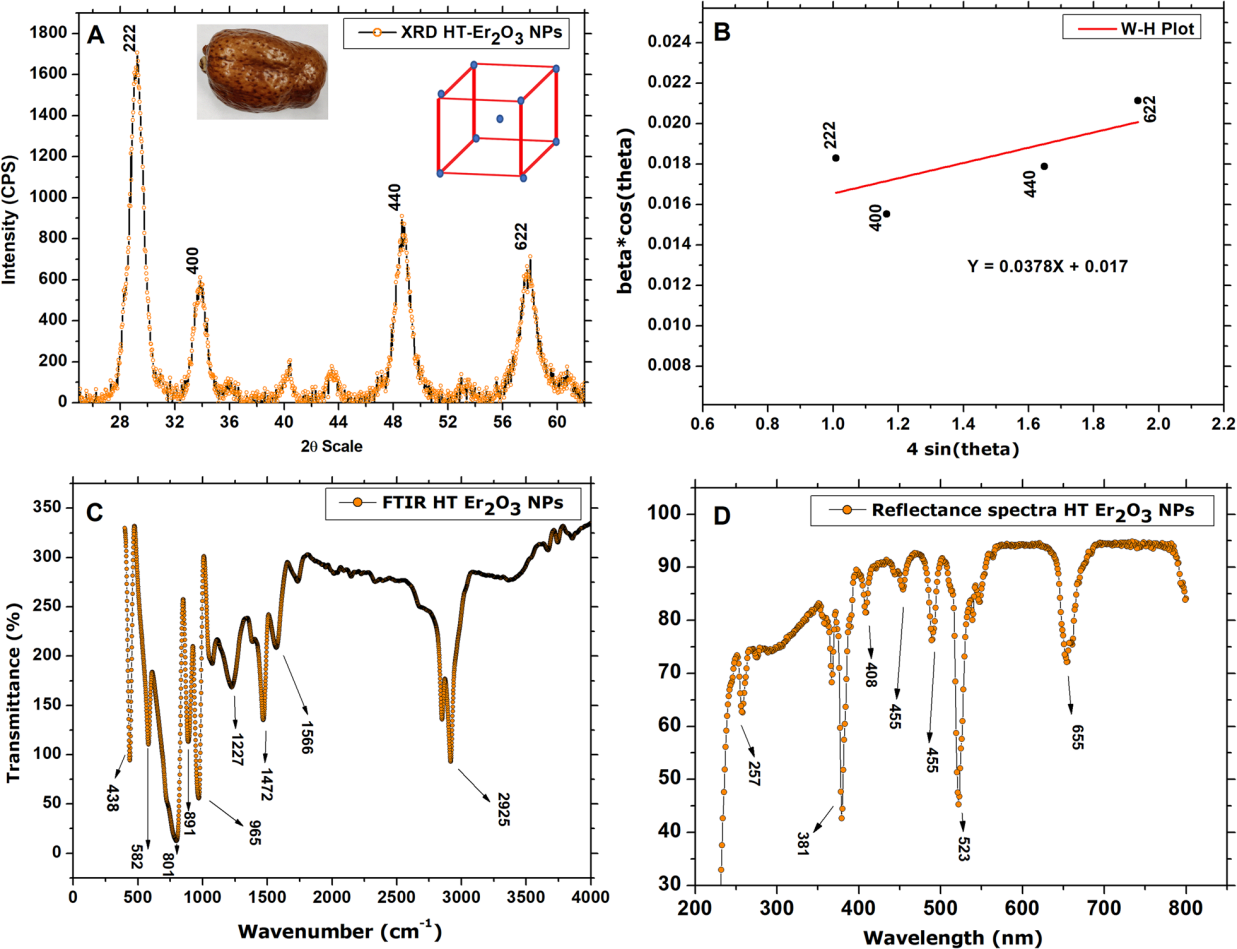
Room temperature physical properties of the HT-Er_2_O_3_ NPs; (**A**) X-ray diffraction pattern; (**B**) Williamson Hall Plot analysis of the HT-Er_2_O_3_ NPs (From the linear fit, data is extracted to calculate the strain from the slop and crystallite size from the y-intercept); (**C**) FTIR spectra of HT-Er_2_O_3_ NPs; (**D**) Reflectance spectra of HT-Er_2_O_3_ NPs

The room temperature physico-chemical attributes of the HT-Er_2_O_3_ NPs were studied using XRD, FTIR spectroscopy, Reflectance spectroscopy as indicated in the inset Fig. 2(A–D). The X-ray diffraction pattern revealed sharp peaks for 2θ position at 29°, 33°, 48° and 57.9° which can be assigned to the crystallographic reflections of (222), (400), (440) and (622). These results are perfectly in accord with the JCPDS card no. 00-043-1007, that corresponds to the Er_2_O_3_ with body centered cubic crystal lattice. The average crystallite or grain size of HT-Er_2_O_3_ NPs calculated according to the Debye Scherrer approximation (Eq. 1) was found to be ~ 7.56 nm. Obtained calculations are shown in Table 1. The crystallite or grain size calculated from the W–H plot method was found to be 11 nm using (Eq. 2). The results are indicated in Fig. 2B. The dislocation density was calculated as 0.

**Table 1:** Grain or crystallite size of the HT-Er_2_O_3_ NPs

2θ	hkl	FWHM	Grain or crystallite size (nm)
222	222	1.16335	7.37
400	400	1.06846	8.13
440	440	1.1669	7.79
622	622	1.35403	6.98
Average grain or crystallite size			D = 7.56 nm
Dislocation density			δ = 1/D^2^ = 0.0174 nm^−2^
Grain or crystallite size using Williamson-Hall method			11 nm
Dislocation density			δ = 1/D^2^ = 0.008 nm^−2^
Strain (ε)			0.0378

**Fig. 3:**
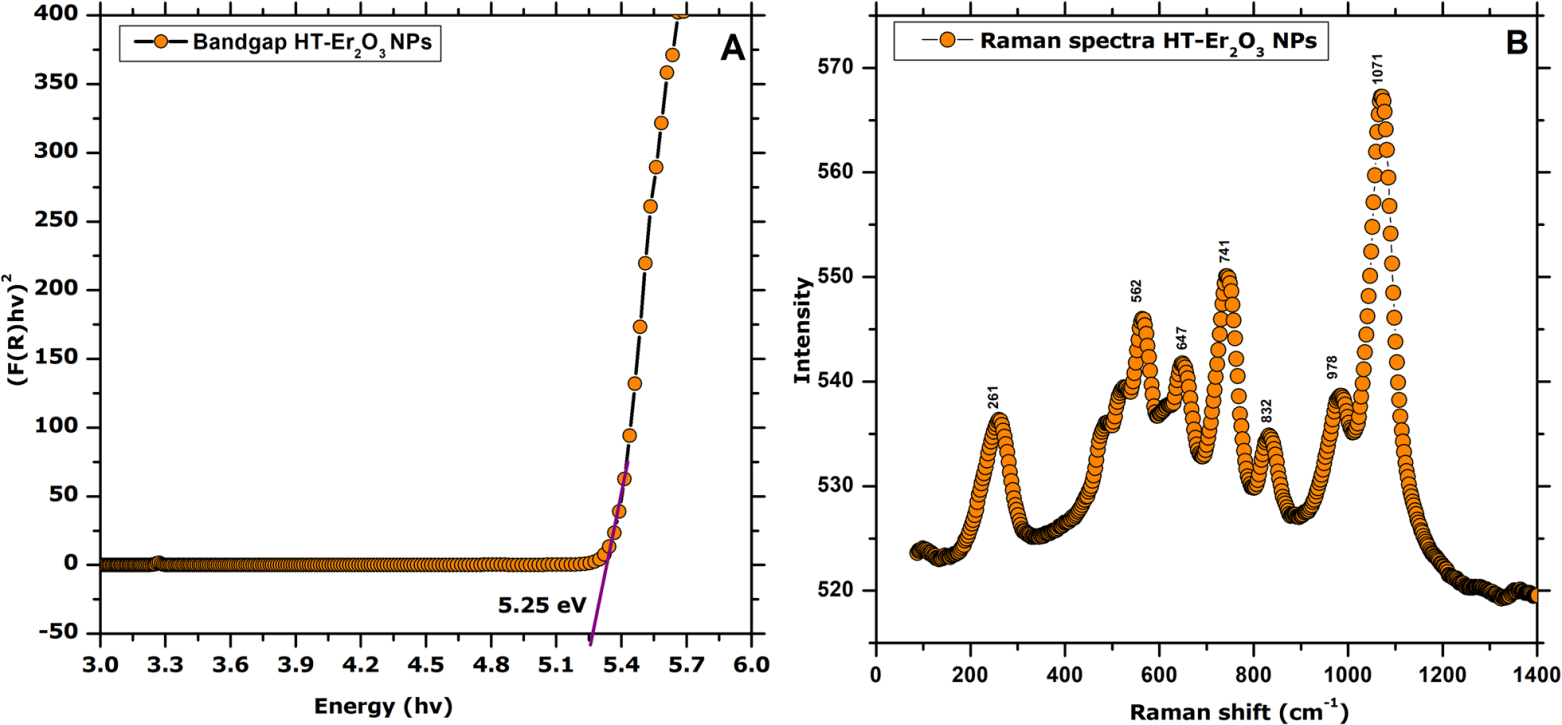
Bandgap calculation and Raman Spectra (**A**) Plots of (F(R)hν)^2^ versus the photon energy (hν) after applying K–M function for calculated the optical bandgap; (**B**) Raman spectra of HT-Er_2_O_3_ NPs

Figure 2C indicates the FTIR spectra of the HT-Er_2_O_3_ NPs, for evaluation of their vibrational properties to understand their structural and functional nature. The FTIR spectra reveals typical metal oxide vibrations in the fingerprint region. Peaks positioned at ~ 438 cm^−1^ and 582 cm^−1^ can be assigned to the Er-O and Er-O-Er, that confirms the synthesis of erbium oxide nanoparticles. The vibrations between 800 cm^−1^ and 3000 cm^−1^ can be ascribed to the specific surface activities, attached functional groups, surface adsorbed moisture or other phenolic compounds. The IR bands can be assigned to different functional groups like ~ 801 cm^−1^ (amine group), 891 cm^−1^ (C–H vibration from aromatic compounds), 965 cm^−1^ (O–H vibration), 1227 cm^−1^ (C–O vibration), 1472 cm^−1^ (C–H bending mode of alkanes), 1566 cm^−1^ (C–C stretching of aromatic compounds), 2925 cm^−1^ (C–H stretching of alkanes). Figure 2D indicates reflectance spectra which was used to derive the optical bandgap (5.25 eV) using Kubelka–Munk function (K–M), i.e., by plotting (F(R)hv)^2^ on y-axis against hv on x-axis, as indicated in Fig. 3A. The K-M function can be determined by using the following formula;

4 $$FR = {\raise0.7ex\hbox{${\left( {1 - R} \right)^{2} }$} \!\mathord{\left/ {\vphantom {{\left( {1 - R} \right)^{2} } {2R}}}\right.\kern-0pt} \!\lower0.7ex\hbox{${2R}$}}$$

here, “R” is the reflectance, “FR” is the Kubelka–Munk function.

Raman spectrum revealed (100 cm^−1^–1400 cm^−1^) revealed seven major peaks centered at ~ 261 cm^−1^, 562 cm^−1^, 647 cm^−1^, 741 cm^−1^, 832 cm^−1^, 978 cm^−1^ and 1071 cm^−1^ as indicated in Fig. 3B. Between 240 and 300 cm^−1^ there are an isolated group of three lines (2 A_g_ + B_g_) is observed to which no Raman line corresponds in A -type spectra. These bands are assigned to the (2 A_g_ + B_g_) modes deriving from (A_2u_ + E_u_) infrared active modes and involving Er-O bonds. Between 350 and 600 cm^−1^, the last group of Raman lines would correspond to the splitting of the A_1g_ + E_g_ stretching vibrations the frequencies of which are very close in the cubic phase. Peaks between 800 and 1200 cm^−1^ are assigned to different functional groups coming from the *H. thebaica* extract which confirmed as well with FTIR.

**Fig. 4:**
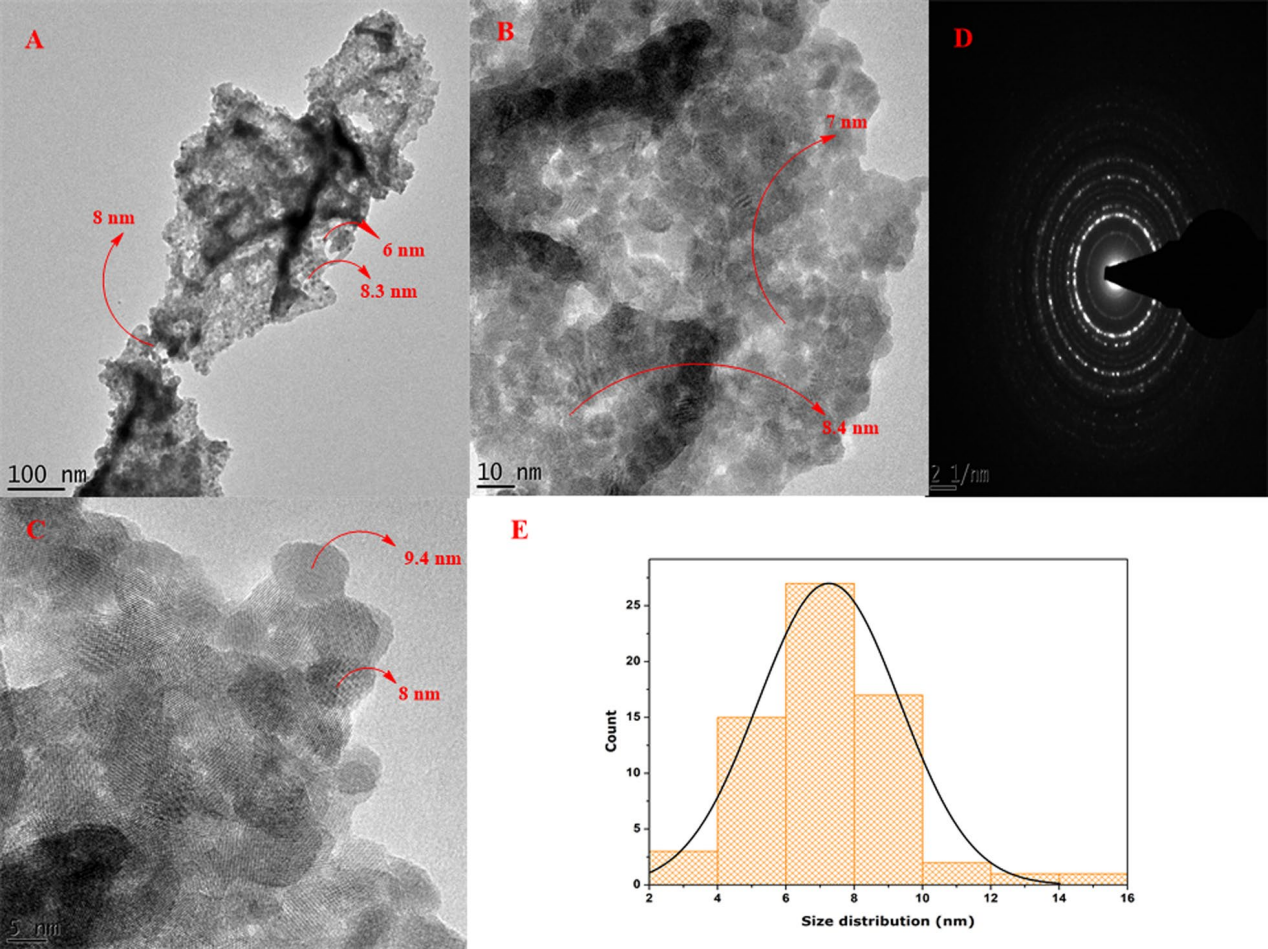
High resolution transmission electron microscopy of HT-Er_2_O_3_ NPs; (**A**–**C**): HR-TEM images at different magnifications; (**D**): Selected Area Electron Diffraction pattern of HT-Er_2_O_3_ NPs; (**E**) Particle size distribution of the HT-Er_2_O_3_ NPs

HR-TEM images at different magnifications were used to identify shape and obtain the size distribution as indicated in the inset Fig. 4A–C. The nanoparticles appeared to be cuboidal and quasi-spherical (Figure A–C), whereas, the size distribution results histogram revealed that most of the nanoparticles were around the range of 6–8 nm (Fig. 4E). The selected area electron diffraction pattern as indicated in Fig. 4D, revealed spotty rings which reaffirmed that the HT-Er_2_O_3_ NPs are of highly crystalline nature.

### α-glucosidase inhibition

**Fig. 5:**
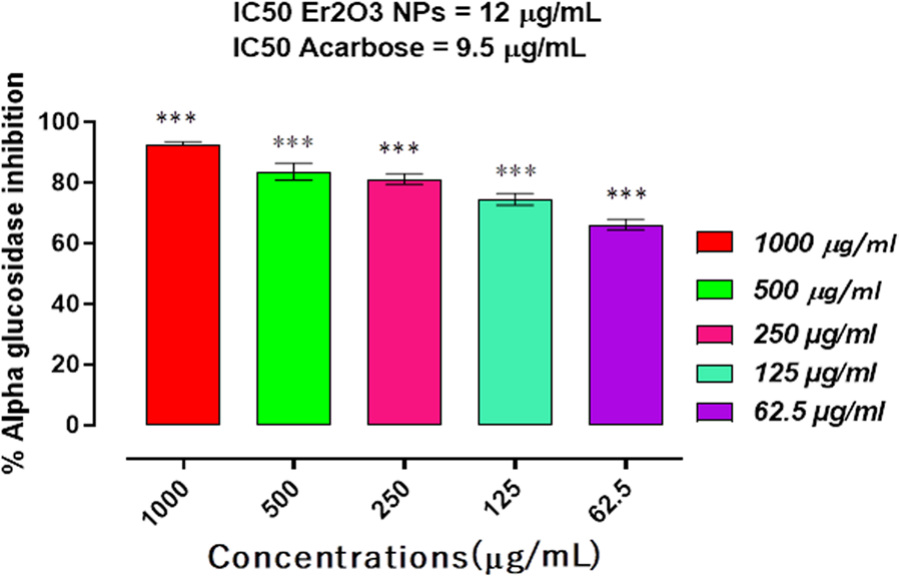
Results of Er_2_O_3_ NPS inhibitory potentials against α-glucosidase enzyme. Results were presented as mean ± SEM of three independent experimental observations. *** represents p value < 0.001 when compared with placebo group. Acarbose was used as positive control

The synthetic HT-Er_2_O_3_ NPs exhibited concentration-dependent inhibitory potentials against alpha-glucosidase enzyme. Results of the standard drug acarbose were comparable as shown in Fig. 5. The percent inhibition against α-glucosidase by HT-Er_2_O_3_ NPs at the different test concentrations in the range of (1000 μg mL^−1^ to 62.5 μg mL^−1^) were 92.30, 83.60, 81.15, 74.43 and 66.13 percent respectively with IC_50_ value of 12 μg mL^−1^. The positive control, acarbose displayed the IC_50_ values of 9.5 μg mL^−1^.

### HT-Er_2_O_3_ NPs inhibit α-amylase enzyme

**Fig. 6:**
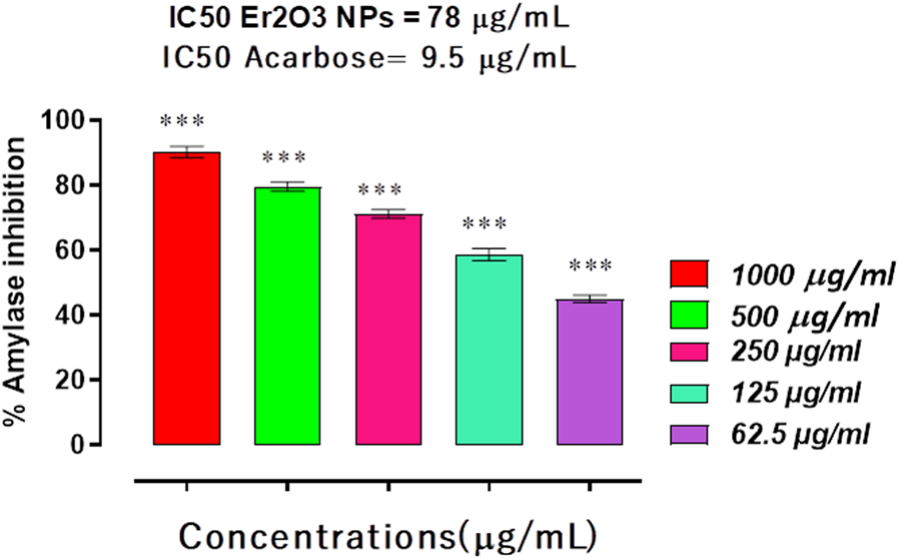
Results of Er_2_O_3_ NPS inhibitory potentials against α-amylase enzyme.Results were presented as mean ± SEM of three independent experimental observations. *** represents p value < 0.001 when compared with placebo group

Again HT-Er_2_O_3_ NPs exhibited considerable inhibition against α-amylase enzyme in comparison to control drug as shown in Fig. 6. The observed percent inhibitions by HT-Er_2_O_3_ NPs were 90.30, 79.63, 71.20, 58.67, and 45.00 at concentrations of (1000 μg mL^−1^ to 62.5 μg mL^−1^), respectively with IC_50_ value of 78 μg mL^−1^. The IC_50_ value of positive control, acarbose was 9.5 μg mL^−1^.

### HT-Er_2_O_3_ NPs scavenge DPPH radicals

**Fig. 7:**
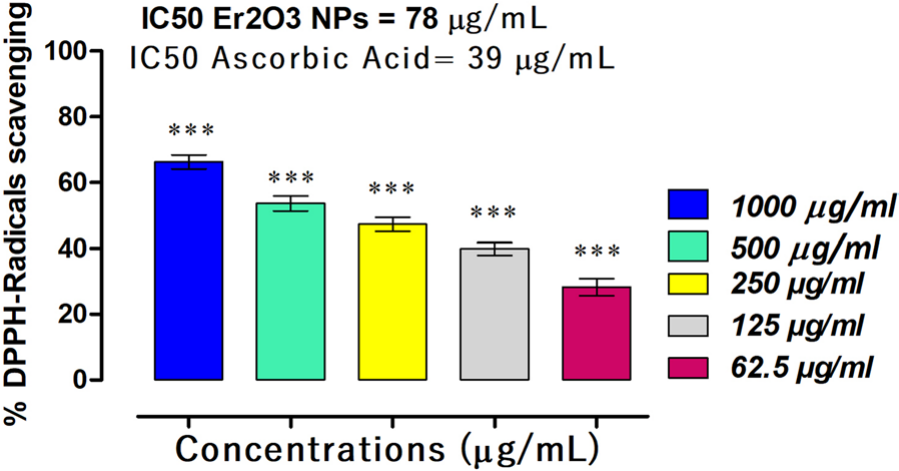
Results of Er_2_O_3_ NPS DPPH radical scavenging potential. *** represents p value < 0.001 when compared with positive control group

In this assay, the synthesized HT-Er_2_O_3_ NPs exhibited high percent activity against DPPH free radicals with concentration dependent manner (Fig. 7). The synthesized HT-Er_2_O_3_ NPs showed 66.25, 53.63, 47.23, 39.82 and 28.31 percent scavenging at the concentrations of (1000 μg mL^−1^ to 62.5 μg mL^−1^) respectively with IC_50_ of 78 μg mL^−1^. The % DPPH scavenging of HT-Er_2_O_3_ NPs was compared with Ascorbic acid (positive control), whose IC_50_ value was 39 μg mL^−1^.

### Acute toxicity test

In acute toxicity study, no animals’ lethality was observed for one week. Further, no cyanosis, spontaneous activity, tail pinch, aggressiveness, convulsions or bizarre behavior was observed during the course of study. So, our sample was observed to be safe at the tested concentrations, though its chronic effects on individual organs need further detailed studies.

### OGTT results

**Fig. 8:**
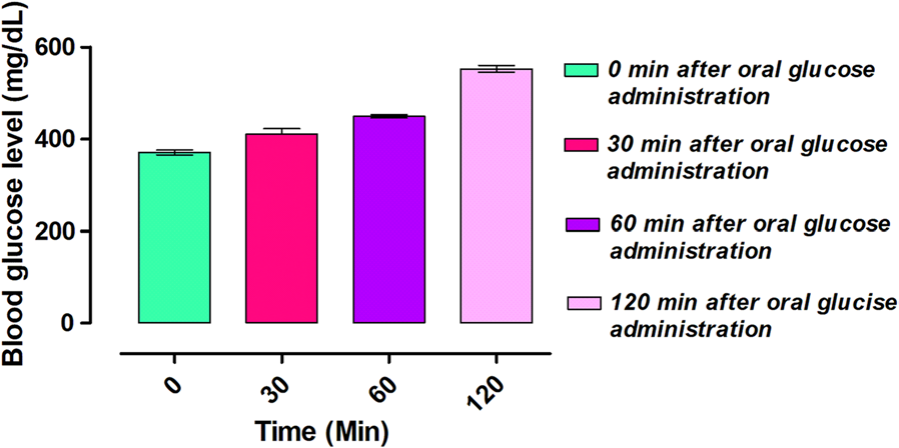
Results of the oral glucose tolerance test (OGTT)

After induction of diabetes and administration of oral dose of glucose the blood glucose was 370.66 μg dL^−1^ at 0 time, 411.00 μg dL^−1^ after 30 min, 449.33 μg dL^−1^ at 60 min and 552.66 μg dL^−1^ after 120 min. Results of the assay is indicated in Fig. 8.

### HT-Er_2_O_3_ NPs exhibited considerable anti-hyperglycemic effects

Results of various treatments on blood glucose level of animals is summarized in Table 2. Briefly, in disease group (group 1) the blood glucose level was observed to be elevated persistently 567.00 ± 3.46 mg dL^−1^ on day 0, till 389.33 ± 4.50 mg dL^−1^ on day 21. Blood glucose level of saline treated group (group 2) was within the range throughout the study. Though it was little elevated 115.33 ± 7.86*** mg dL^−1^ l at the start of experiment but then stabilized and on day 21 it was 93.50 ± 1.60*** mg dL^−1^. Positive control, group (group 3) maintained the blood glucose level of diabetic animals in an average range of (376.66 ± 1.20*** mg dL^−1^ on day 0, 351.33 ± 5.20*** mg dL^−1^on day 1, 341.00 ± 3.66*** mg dL^−1^ on day 4, 301.38 ± 2.33*** mg dL^−1^ on day 11, 279.88 ± 3.80*** mg dL^−1^ on day 14 and 235.33 ± 2.33*** mg dL^−1^ on day 21. Overall glibenclamide was unable to control hyperglycemia in diabetic animals. Our tested compound HT-Er_2_O_3_ NPs have exhibited better control over blood glucose level of the diabetic animals (503.66 ± 5.92*** on day 0 and 185.66 ± 2.60*** on day 21).

**Table 2:** Effect of various treatments on blood glucose levels of diabetic animal

S. No.	Groups	Dose mg kg^−1^	Blood glucose level (GL) (mg dL^−1^)						
			Day 0	Day 1	Day 4	Day 7	Day 11	Day 14	Day 21
Group1	After Alloxan + Tween 80	150	567.00 ± 3.46	507.00 ± 5.77	522.66 ± 3.75	500.66 ± 4.33	469.66 ± 2.60	454.33 ± 2.96	389.33 ± 4.50
Group2	Normal control saline	0.35	115.33 ± 7.86***	121.00 ± 3.51***	116.50 ± 2.66***	108.33 ± 2.89***	103.00 ± 4.50***	97.00 ± 1.50***	93.50 ± 1.60***
Group3	Glibenclamide	05	376.66 ± 1.20***	351.33 ± 5.20***	341.00 ± 3.66***	361.50 ± 2.50***	301.38 ± 2.33***	279.88 ± 3.80***	235.33 ± 2.33***
Group4	HT-Er_2_O_3_ NPs	30	503.66 ± 5.92***	458.66 ± 2.02***	375.00 ± 3.09***	310.66 ± 2.33***	298.00 ± 4.04***	205.33 ± 2.60***	185.66 ± 2.60***

^***^p < 0.001 at 95% confidence interval

### Biochemical analysis of blood

**Fig. 9:**
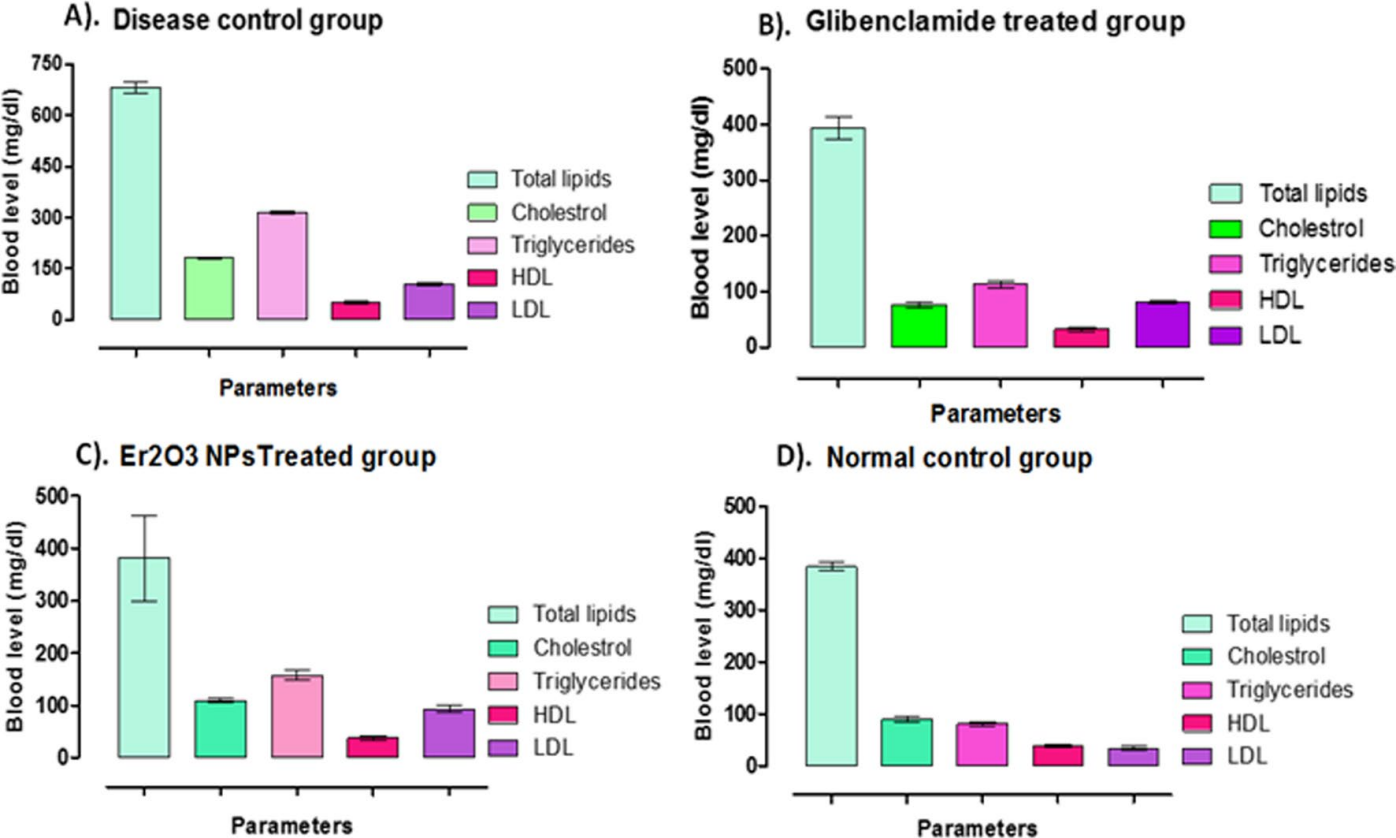
Effect of Er_2_O_3_ NPs therapy on serum lipids including total lipids, cholesterol, triglycerides, HDL and LDL. (**A**): Disease group; (**B**): Positive control group, (**C**): Er_2_O_3_ NPs treated group; (**D**): Normal group

Results of the biochemical analysis of blood is summarized in the inset Fig. 9A–D. In disease group, average total lipids were 682.33 μg dL^−1^, cholesterol level was 180.66 μg dL^−1^, triglycerides were 315.00 μg dL^−1^, HDL was 51.33 and LDL was 105 mg/dl respectively (Fig. 9A). Treatment with HT-Er_2_O_3_ NPs have normal blood parameters i.e. total lipids, cholesterol, triglycerides, HDL and LDL after 21 days as observed in Fig. 9C. The blood glucose level was at comparatively low level in the positive control group indicating reduction in the hyperglycemia mediated changes in the blood total lipids, triglycerides, cholesterol, HDL and LDL levels (Figure C).

## Discussion

Biological synthesis of the metal NPs provides an eco-friendly and one-step process for the synthesis of novel metal nanoparticles. Previously, we have demonstrated synthesis of Fe_2_O_3_ (Mohamed et al. 2020b), CeO_2_ (Mohamed et al. 2020a), Cr2O_3 _ (Mohamed et al. 2020), BiVO_4_ (Mohamed et al. 2019b), ZnO (Mohamed et al. 2020c), CuO (Mohamed et al. 2021) and Ag nanoparticles (Mohamed et al. 2019a) using the fruit material of *Hyphaene thebaica* fruit material. To date, the exact mechanism of bioreduction leading to the synthesis of metal nanoparticles is not well explored because of the heterogeneity of the plant extracts (Dauthal and Mukhopadhyay 2016). Plant phytochemicals possess different chemical and physical properties including metal ion reduction and stabilization (Abomuti et al. 2021). The generally proposed mechanism includes a simple bioreduction and stabilization process which is mediated by the phytochemical constituents in the plant extracts. Phytochemicals plays a dual role, thereby, at first reduces the metal salt in to ions, in this case reducing the erbium nitrate, followed by stabilization through the capping or coating agents found in the fruit extracts of the *Hyphaene thebaica.* The intricate nature and phytochemistry of the plant extracts makes it difficult to specify a particular class of specific biomolecules in metal reduction, stabilization and morphological control of NPs (Huo et al. 2022).

Here, the green synthesis of nano-erbia is reported for the first time in the literature. Medicinal plants like *H. thebaica* have a rich phytochemistry and includes compounds like vanillic acid, cinnamic acid, sinapic acid, caffeic acid, chlorogenic acid, epicatechin, hesperetin, naringin, glycosides, quercetin, rutin, tannins, saponins etc. Such phenolic and flavonoid compounds have the ability to execute redox reactions and subsequently stabilize the nanoparticles (El-Beltagi et al. 2018; Islam et al. 2022; Jadoun et al. 2021; Jeevanandam et al. 2022; Karatoprak et al. 2017). Previous studies have indicated that the phenolic compounds possess strong reducing ability and hence most likely agents for catalyzing the bioreduction as compared to other macromolecules in plant extracts like carbohydrates and proteins (Huo et al. 2022).

X-rays diffraction pattern was found to be consistent with the previously published literature (Munawar et al. 2020; Wang et al. 2018b). Similarly, the FTIR peak at ~ 438 (Er-O) cm^−1^ and ~ 582 cm^−1^ (Er-O-Er) are in agreement with reported literature (Azad and Maqsood 2014). The FTIR signature indicated that different phytochemicals available in the pristine aqueous fruit extracts were responsible for the reduction and capping process and eventually led to the synthesis of stable nanoparticles (Khan et al. 2013). The optical bandgap of the HT-Er_2_O_3_ NPs was calculated 5.25 eV by applying the K–M function which is correlates with values reported in the literature (Kamineni et al. 2012; Kao et al. 2012). The Raman vibrations corelates with the previous studies (Joya et al. 2019).

Diabetes mellitus (DM) is a disorder of metabolism of glucose that encompasses raised levels of blood glucose and other complications like neuropathy, retinopathy, nephropathy, and micro and macrovascular complications (Arky 1982). Defects in the production, secretion and action of insulin are responsible for hyperglycemia (Booth et al. 2016). DM affects about five percent population worldwide and its occurrence is increasing at an alarming rate, and is also associated with number of other diseases (Rahim et al. 2019). There are about 450 million peoples around the world which are effected by DM and the numbers are expected to grow up to 690 million by 2044 (Cho et al. 2018). Type 1 (T1DM) and Type 2 (T2DM) are the important kinds of DM. (Del Prete et al. 1977; Himsworth and Kerr 1939). Type 1 diabetes is an immune-associated, destruction of insulin-producing pancreatic β cells (Atkinson et al. 2011). Type 1 diabetes treatment includes the use of insulin and its analogues. T2DM can be managed by controlling diet, obesity and with anti-diabetic drugs (Ohlson et al. 1985). In the development of antidiabetic therapeutic agents, α–amylase and α–glucosidase inhibitors are the vital targets. These enzymes breakdown polysaccharides (starch) to glucose and the inhibition of these enzymes play an important role to reduce glucose absorption in the intestine (Gin and Rigalleau 2000). There are several therapeutic approaches used for the treatment of diabetes, like lowering postprandial hyperglycemia by hydrolyzing α-amylase and α-glucosidase enzyme inhibition (Wang et al. 2018a). Anti-diabetic drugs as enzyme inhibitors like acarbose, voglibose, and miglitol were utilized for years to treat diabetes (Chaudhury et al. 2017).

Plants contains numerous secondary metabolites exhibiting different properties including the metal ion reduction and stabilization. The interaction of plants with metals as a natural process and have got the attention of many scientists to utilize the secondary metabolites of plants as a reducing and stabilizing agent (Javed et al. 2021). The biosynthesis of metal NPs utilizing medicinal plants serve as alternative to utilizing hazardous chemical and physical synthetic techniques (Anand et al. 2017). We for the first-time synthesized HT-Er_2_O_3_ NPs by using plant extracts as reducing and stabilizing agents. The prepared nanoparticles were extensively characterized in their in-vitro and in-vivo assays were performed for determination of its antioxidant and antidiabetic potential.

The synthetic HT-Er_2_O_3_ NPs exhibited concentration dependent inhibition against α-glucosidase and the percent inhibition at the concentrations of 1000, 500, 250, 125 and 62.5 μg mL^−1^ was 92.30, 83.60, 81.15, 74.43 and 66.13 percent respectively with IC_50_ value of 12 μg mL^−1^. In α-amylase inhibition assay, HT-Er_2_O_3_ NPs showed 90.30, 79.63, 71.20, 58.67, and 45.00 percent inhibition at concentrations of 1000, 500, 250, 125, and 62.50 μg mL^−1^, respectively with IC_50_ value of 78 μg mL^−1^. In DPPH free radical scavenging assay, the synthesized HT-Er_2_O_3_ NPs showed 66.25, 53.63, 47.23, 39.82 and 28.31 percent inhibition at the concentrations of 1000, 500, 250, 125 and 62.5 μg mL^−1^ respectively with IC_50_ of 78 μg mL^−1^ (Fig. 1). Animal studies revealed that our tested sample are safe at higher doses and was effective in lowering post-prandial hyperglycemia. As summarized in Table 2, our tested compound HT-Er_2_O_3_ NPs have exhibited better control over blood glucose level of the diabetic animals (503.66 ± 5.92*** on day 0 and 185.66 ± 2.60*** on day 21 as compared to standard drug glibenclamide. Er_2_O_3_ NPs therapy for 21 day has normalized hyperlipidemia. Thus in-vitro enzyme inhibition coupled with anti-radical properties and in-vivo anti-hyperglycemic and anti-hyperlipidemic potentials make it a potential candidate for further detailed studies.

Our findings suggest that our synthetic HT-Er_2_O_3_ NPs have considerable antidiabetic potential via α–glucosidase and α–amylase enzymes inhibition which are the potential targets in the development of antidiabetic drugs. Considerable in-vivo anti-hyperglycemic and anti-hyperlipidemic potentials were observed for HT-Er_2_O_3_ NPs and thus it warrants further detailed studies.

## Data Availability

All the data generated as a result of this current research has been included in the manuscript.
